# Current endometriosis care and opportunities for improvement

**DOI:** 10.1530/RAF-22-0091

**Published:** 2023-08-02

**Authors:** Charlotte Pickett, Warren G Foster, Sanjay K Agarwal

**Affiliations:** 1 Department of Obstetrics, Gynecology and Reproductive Sciences, Center for Endometriosis Research and Treatment, UC San Diego, La Jolla, California, USA; 2 McMaster University Health Sciences Center, Hamilton, Ontario, Canada

**Keywords:** endometriosis, chronic care model, chronic pelvic pain, healthcare costs, multimodal management

## Abstract

Endometriosis is a chronic disease associated with debilitating pain that affects many people assigned female at birth, from menarche through menopause, not just causing pain and infertility but also negatively impacting quality of life, participation in daily activities, productivity, and income. It is associated with increased incidence of obstetric and neonatal complications, anxiety, other chronic diseases, and substantial healthcare costs. Despite the profound negative impact of endometriosis on quality of life, current treatment options remain suboptimal and many patients express dissatisfaction with current care. The prevailing acute-care, single-provider model in which the provider works in relative isolation and thus with limited diagnostic and therapeutic strategies readily available proves inadequate for treating endometriosis. Patients would benefit from earlier diagnosis and referral to a center capable of providing a comprehensive and multimodal management plan that utilizes a chronic care model. Often this can only be achieved through multidisciplinary teams of providers with expertise in endometriosis. The authors acknowledge that many low- and middle-income countries do not have the resources to support such centers but could still benefit from any breakthroughs in treatment they bring about. Researchers need to agree on standardized core outcome measures, relevant to patients with endometriosis and the healthcare system as a whole. Only through increased societal and healthcare provider education and recognition of endometriosis as a chronic disease can we achieve better treatment outcomes.

**Lay summary:**

Endometriosis is a disease that affects about one out of every women. It occurs when tissue like that which is normally located inside the uterus is present outside the uterus. The body’s reaction to this tissue causes inflammation and pain, usually so severe that it disrupts daily activities. Our current medical system does not serve these patients well. Patients with endometriosis often must see many different doctors over many years before learning of their disease and getting treatment. We need to increase awareness of endometriosis and think of it as a chronic disease like diabetes or heart disease. We can improve care by creating centers where experienced teams work together to treat patients and study treatment impacts on quality of life. It is time to adopt a new model for caring for patients with endometriosis.

## Introduction

Endometriosis is a chronic disease associated with debilitating pain that affects at least 6–10% of reproductive age women and people assigned female at birth, independent of whether they identify as female (Fuldeore & Soliman 2017). Studies suggest that many more go undiagnosed (Ferrero *et al.* 2010). The disease is estrogen dependent, inflammatory in nature, and defined by the presence of endometrial like tissue located outside the uterus (Horne & Missmer 2022). It most commonly affects individuals from menarche through menopause, though it can also affect adolescents and postmenopausal women. It is a common cause of pain and infertility but also negatively impacts quality of life, intimate relationships, participation in daily activities, social activity, productivity, and income (Missmer *et al.* 2021). It is associated with increased incidence of obstetric and neonatal complications, depression, other chronic diseases, and substantial healthcare costs (Kvaskoff *et al.* 2015, Soliman *et al.* 2017, Zullo *et al.* 2017). Endometriosis treatment care models remain suboptimal. Many patients express ongoing pain and reduced quality of life even with access to tertiary-care centers (De Graaff *et al.* 2013).

## Current model of care

Extensive data have demonstrated that the currently prevailing acute-care, single-provider model in which the provider works in relative isolation and thus with limited therapeutic strategies readily available proves inadequate in treating endometriosis (Sinaii *et al.* 2007, De Graaff *et al.* 2013, Soliman *et al.* 2016). This is despite significant healthcare resources being directed at the disease (Soliman *et al.* 2016). Extensive data are available indicating that real-world clinical outcomes of patients with endometriosis remain unacceptable. For example, an estimated 70% of patients with endometriosis experience unresolved pain despite the substantial healthcare utilization (Sinaii *et al.* 2007, De Graaff *et al.* 2013). Ferrero *et al.* evaluated a population of 1291 women seeking consultation from their general practitioner for a nongynecologic problem and found that only 28 (2.1%) had a prior diagnosis of endometriosis. A simple questionnaire investigating the presence of dysmenorrhea, dyspareunia, chronic pelvic pain, and dyschezia initiated workup and diagnosis of an additional 46 (3.6%) people with the disease (Ferrero *et al.* 2010). Studies such as this demonstrate the overall poor awareness of endometriosis even among healthcare providers and that simple screening by the healthcare team can identify patients suffering without their provider knowing. Indeed, patients successfully diagnosed with endometriosis experience a delay from symptom onset to diagnosis ranging from 4 to 11 years (Nnoaham *et al.* 2011). This delay in diagnosis affects patients globally, even in countries with universal healthcare and endometriosis centers of excellence, suggesting a lack of recognition of clinical symptoms on the part of patients and primary care providers (Hudelist *et al.* 2012, Ghai *et al.* 2020). It appears that ‘normalization’ of symptoms and misdiagnosis further contribute to delays (Nnoaham *et al.* 2011). Others argue that a lack of clear clinical criteria for diagnosis and reliance on laparoscopy contributes substantially to delays in care (Agarwal *et al.* 2019*a*, As-Sanie *et al.* 2019). By increasing patient and healthcare provider awareness of endometriosis together with the development of reliable noninvasive diagnostic tests, it is possible that the delay in diagnosis can be reduced and initiation of therapy expedited.

## Treatment limitations

Once diagnosed with endometriosis, patients face suboptimal long-term management options. Because endometriosis is an estrogen-dependent and inflammatory disease that is primarily treated by suppressing estrogen and ovulation, many are forced to decide between managing pain and fertility. Combined oral contraceptive pills are often considered first-line treatment (Brown *et al.* 2018, Falcone & Flyckt 2018). For those unable to tolerate estrogen or with contraindications, progestin-only hormonal options are often tried, though progestin resistance may develop (Joshi *et al.* 2017). Progesterone receptor expression may predict clinical response and so has the potential to advance personalized medicine for endometriosis (Flores *et al.* 2018). Gonadotrophin-releasing hormone (GnRH) agonist and antagonist therapies are usually considered next. These therapies have proven effective for pain relief, improved quality of life, and productivity. However, they come with many of the side effects associated with menopause, and there is some concern that GnRH antagonists in particular could worsen preexisting mood disorders. Both are only approved for relatively short-term use due to concerns regarding decreased bone mineral density (Surrey *et al.* 2018, 2019). Clearly, this is inadequate for a chronic disease. Further, medical treatment is usually suppressive not curative, with symptoms typically recurring quickly after therapy discontinuation.

Data regarding the efficacy of surgical excision or ablation of endometriosis for pain management are inconclusive (Bafort *et al.* 2020). This is largely because the best surgical approach for endometriosis is controversial and operator dependent. Heterogeneity often precludes meta-analysis of studies due not just to surgeon skill but techniques employed (ablation, excision, and laser), the extent of the disease removed, whether hysterectomy or oophorectomy was performed, and use of postoperative medical suppression. Studies often lack long-term follow-up and risk publication bias. Additionally, the American Society of Reproductive Medicine classification system, the most widely used to stage endometriosis, is poorly correlated with surgical complexity or pain symptoms (Andres *et al.* 2018, Abrao *et al.* 2021). The ENZIAN classification system was introduced in 2005 to better classify deeply infiltrative endometriosis and is advantageous because it allows for classification based on either surgery or imaging. However, it has a poor level of international acceptance and is quite complex (Keckstein *et al.* 2021). The AAGL classification system has just recently been introduced and better addresses surgical complexity but is also poorly correlated with pain scores, consistent with the wide variation in disease severity observed among patients presenting with pain (Fauconnier *et al.* 2002, Mak *et al.* 2022). In patients with chronic pelvic pain undergoing excision of all visible endometriosis without hysterectomy, need for reoperation is common (~20% at 2 years and 58% at 7 years) (Shakiba *et al.* 2008). Hysterectomy decreases the risk of reoperation at 7 years to 24% and with bilateral oophorectomy the risk drops to less than 8% (Shakiba *et al.* 2008). Of course, the latter operations compromise fertility, and one must weigh the risks of increased cardiovascular disease and all-cause mortality with early surgical menopause (Rush *et al.* 2022). Even in the hands of expert surgeons, excision of deep infiltrating endometriosis requiring segmental bowel resection is associated with significant surgical complications (Roman *et al.* 2018).

## Poor real-world outcomes

Within the existing treatment paradigm, real-world outcomes for patients with endometriosis suffer despite the high cost of care. This inadequacy in care is partially reflected in the number of emergency department visits that occur each year for endometriosis, which did not decline from 2006 to 2015, despite increased charges per visit (Agarwal *et al.* 2020). The estimated direct cost of endometriosis in the United States is $12,118 per patient per year (Soliman *et al.* 2016). Soliman *et al.* in a 2018–2019 cross-sectional survey of nearly 30,000 Canadian patients demonstrated that those with self-reported endometriosis have significantly lower quality-of-life scores than those without endometriosis. Interestingly, disease impact on their mental health was greater than that on their physical health (Soliman *et al.* 2020). This could be due to high rates of anxiety, depression, and emotional distress associated with the diagnosis of endometriosis, which can lead to social isolation and feelings of hopelessness (Culley *et al.* 2013, Missmer *et al.* 2021**)**. Additionally, while patients reported receiving various therapies for endometriosis, they also noted a high frequency of pain symptoms, consistent with other studies, demonstrating an unmet need for pain relief in patients with endometriosis (Culley *et al.* 2013, De Graaff *et al.* 2013,Missmer *et al.* 2021**)**.

## Moving forward

Our understanding of endometriosis would improve greatly with longer-term assessments of treatment. Because of the chronicity and recurrent nature of endometriosis, the 12-month follow-up period utilized in most studies is highly inadequate. Of course, longer studies require greater funding; however, to date, endometriosis is underresearched and underresourced, perhaps in part due to gender bias in the treatment of pain (Samulowitz *et al.* 2018, As-Sanie *et al.* 2019).

Earlier diagnosis of the disease after symptom onset is imperative to improving life-course potential (Missmer *et al.* 2021). Endometriosis and its associated symptoms have been shown to hamper education attainment, work productivity, career success, social life, personal relationships, mental and emotional health, and quality of life (Missmer *et al.* 2021). Early diagnosis and treatment have the potential to improve the life-course and fertility outcomes while reducing the risk of central sensitization and chronic pain (Stratton & Berkley 2011). It should not take patients an average of seven visits to their primary healthcare provider before being referred to a specialist (Nnoaham *et al.* 2011). Greater education regarding endometriosis is needed at every level, from the public to healthcare students to gynecologists themselves. It has been suggested that clear guidelines on when to initiate empiric treatment vs referral to a specialist would help speed up the referral process. Appropriately, many providers are hesitant to perform or refer for diagnostic laparoscopy, particularly in adolescents and young patients, due to the invasive nature of surgery (van der Zanden *et al.* 2020). However, this need not delay clinical diagnosis and management of symptoms (Chapron *et al.* 2019). Imaging modalities like transvaginal ultrasound and magnetic resonance imaging (MRI) are helpful for diagnosing endometriomas and some cases of deep infiltrating disease but do not reliably detect superficial peritoneal implants (Nisenblat *et al.* 2016). Advances in imaging technique, such as use of bowel preparation with ultrasound and 3.0 Tesla MRI, show promise in improving endometriosis detection rates (Nisenblat *et al.* 2016). A new ultrasound technique involving sterile saline infusion into the peritoneal cavity has shown promise for the detection of superficial disease (Leonardi *et al.* 2020). While newer imaging modalities are encouraging, larger studies are needed to establish their value as replacement tests or triage tests for a laparoscopic diagnosis (Nisenblat *et al.* 2016, Pascoal *et al.* 2022). Research is underway to identify circulating markers predictive of endometriosis, with the aim of early and noninvasive methods for diagnosis. Again, while results are promising (Cosar *et al.* 2016, Anastasiu *et al.* 2020, Moustafa *et al.* 2020, Papari *et al.* 2020), further investigation is needed before they can be recommended in routine practice as a triage test (Pascoal *et al.* 2022).

Once diagnosed, patients with endometriosis may benefit from a comprehensive and multimodal management plan. Often this can only be achieved through multidisciplinary teams of providers with expertise in endometriosis. The gynecologist remains central to diagnosis, patient education, specialty referrals, and long-term follow-up. They can determine, with the patient, the need for collaboration with integrative medicine (acupuncture, nutrition, and mind–body programs), mental health, pain medicine, specialist surgeons, physical therapy, gastroenterology, urology, or other experts (Agarwal *et al.* 2019*b*). Such multidisciplinary treatment approaches address the fact that patients with endometriosis are likely to have co-occurring pain processes such as pelvic floor myalgia, irritable bowel disease, and interstitial cystitis, to name a few. Focus can turn from a single intervention to long-term management, with combination therapies proven to improve outcomes (Zakhari *et al.* 2021). Additionally, such models of care allow for providers to readily recognize and address the impact of the disease on mental health and facilitate patients in developing strategies for managing the stresses inherent to suffering from a chronic, painful illness (Appleyard *et al.* 2020). Research demonstrates a multidirectional relationship between mental health and pain. Thus, stress management techniques and alteration of brain–body–brain pathways are an important therapeutic option for endometriosis (Appleyard *et al.* 2020). Creating centers of excellence also has the potential to consolidate endometriosis surgeries among high-volume surgeons, thereby improving outcomes and lowering complication rates (Mowat *et al.* 2016). Additionally, clinical experience and accurate imaging allow for preoperative planning and collaboration between surgical disciplines. This in turn has the potential to facilitate more comprehensive and effective operations, improving outcomes while minimizing the number of total operations a patient needs during their lifetime.

**Figure 1:**
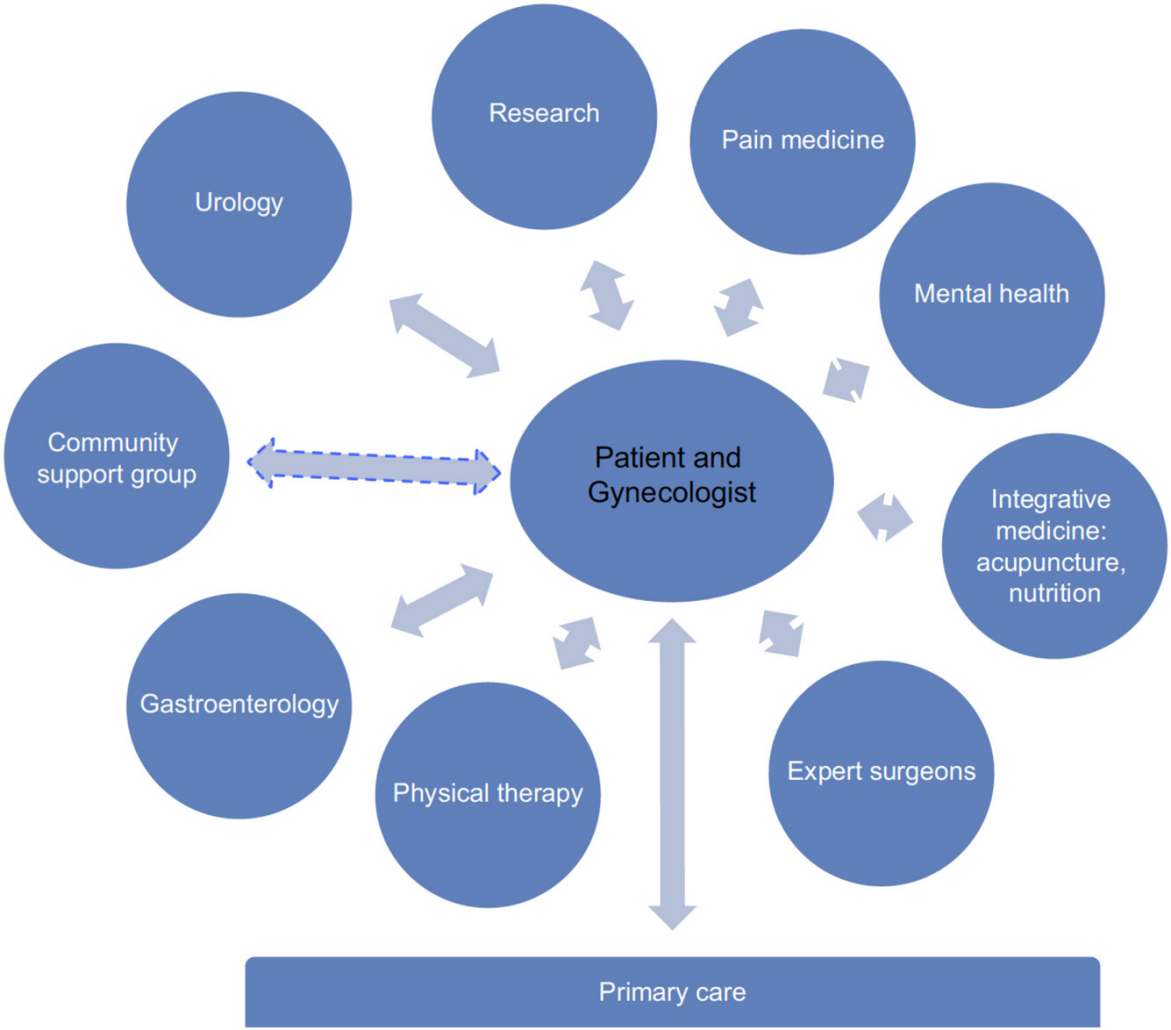
The multidisciplinary endometriosis care model used at our institution (Agarwal *et al.* 2019*b*). The nondashed arrows represent relationships already established within our model and the dashed arrow represents a relationship we are working to establish but have not yet finalized. The figure is reproduced from Agarwal *et al*. (2019*b*) *International Journal of Women’s Health* 2019 11 405-410. Originally published by and used with permission from Dove Medical Press Ltd.;

Models of care capable of providing improved clinical outcomes through this type of multidisciplinary, comprehensive, and patient-focused disease management will inevitably vary by regional healthcare systems. It is our impression that with the establishment of a Center for Endometriosis Research and Treatment (Fig. 1), we have been able to provide a higher level of patient-focused comprehensive endometriosis care (Agarwal *et al.* 2019*b*). This Center was designed around the chronic care model (CCM), which was developed for improving care for individuals with chronic diseases in primary care (Wagner 1998). The provision of multidisciplinary care by a variety of providers that are experts both in their fields and in endometriosis provides hope of a more comprehensive chronic care. Clearly, appropriate health services research evaluating both positives and negatives is required to validate such multidisciplinary care models. Investigation will need to focus not solely on the effectiveness of the various components of multidisciplinary care and the presence or absence of synergy between them but also on predictors of success. In the meantime, implementation of multidisciplinary care models may face barriers including those pertaining to cultural differences, logistics, geographical location, health insurance costs, funding, and the willingness/availability of experts to participate.

Finally, research into effective endometriosis treatment is hampered by widespread variation in outcome reporting and short durations of investigation. We applaud Duffy JMN *et al.* for developing a core outcome set to guide future endometriosis research (Duffy *et al.* 2020). We agree that overall pain, improvement in the most troublesome symptoms, quality of life, adverse events, and patient satisfaction with treatment should be reported in endometriosis research. However, the remainder of the outcomes they identified pertain only to fertility patients. Although standardized outcomes based on input from patients, clinicians, and researchers would be ideal for setting national and international standards, the goals of the particular patient seeking help at the time are what matter most in the clinical setting. Collaboration is needed between researchers and clinicians to conduct large-scale well-designed trials of adequate duration to further guide clinical decision-making.

In conclusion, patients with endometriosis face a debilitating chronic disease. The literature demonstrates that current diagnostic and management strategies inadequately address patient needs. In health systems and locations where feasible, we propose a transition from a single-provider, acute-care and lesion-focused model to one that includes regional multidisciplinary teams of providers focused on treating the patient and their symptoms as a whole and incorporating expertise from various relevant specialties. We predict that comprehensive multidisciplinary care has the potential to provide a broader range of effective interventions than conventional care, which can further improve quality of life and hence the life course of patients suffering from endometriosis.

The responsibility of creating comprehensive endometriosis treatment centers falls not just on individual providers or institutions. Improvements in endometriosis awareness and treatment require the joint effort of medical societies and patient advocacy groups working with government to bring about policy change. Similarly, it is likely only with coordination between our professional organizations, public health associations, and global research funding agencies that we will see better endometriosis research.

## Declaration of interest

All authors declare that there is no conflict of interest that could be perceived as prejudicing the impartiality of the research reported.

## Funding

This research did not receive any specific grant from any funding agency in the public, commercial, or not-for-profit sector.

## Author contribution statement

WF and SA conceived this review and wrote the outline. CP wrote the first draft of the paper. WF and SA added to and edited the paper.
